# Lymphoid interstitial pneumonitis in a newly diagnosed late presenter of human immunodeficiency virus infection: a case report

**DOI:** 10.1186/s13256-020-02572-w

**Published:** 2021-02-02

**Authors:** V. Petrakis, P. Panagopoulos, P. Ntolios, I. Chrysafis, M. Georgaraki, D. Papazoglou

**Affiliations:** 1 HIV Unit, University General Hospital Alexandroupolis, Democritus University of Thrace, Xanthi, Greece; 2 Respiratory Medicine Department, University General Hospital of Alexandroupolis, Alexandroupoli, Greece; 3 Department of Radiology, University General Hospital of Alexandroupolis, Alexandroupoli, Greece

**Keywords:** Lymphoid interstitial pneumonitis, LIP, Late presenters, HIV, AIDS

## Abstract

**Background:**

An increase has been described throughout the years in the frequency of various uncommon diseases in people living with human immunodeficiency virus (HIV). Particularly late presenters are associated with a significant risk not only for acquired immune deficiency syndrome (AIDS)-defining conditions but also for non AIDS-defining diseases which aggravate the prognosis of patients. Lymphoid interstitial pneumonitis (LIP) is one of these conditions described more often after the onset of HIV epidemic. LIP is a benign polyclonal lymphoproliferative disorder of the lung with not well characterized clinical and radiographic findings.

**Case presentation:**

We report the diagnostic approach and clinical progress of a newly diagnosed late presenter of HIV infection with respiratory problems in our HIV unit. The findings of computed tomography indicated the diagnosis of HIV-associated LIP, although this condition is mainly described in a normal range of CD4 cell count.

**Conclusion:**

This case presentation highlights the importance of timely diagnosis and initiation of antiretroviral therapy. The increase of CD4 cell count and viral suppression may improve the symptoms of LIP.

## Introduction

The significant number of late presenters of human immunodeficiency virus (HIV) infection remains a serious public health problem. The annual data demonstrate that we are still far from the termination of HIV epidemic. Approximately half of newly diagnosed people with HIV infection in 2017 in European Countries were late presenters [1]. As late presenters are defined patients presenting for care with a CD4 cell count below 350 cells/mm^3^ or with an acquired immune deficiency syndrome (AIDS)-defining event regardless of the CD4 cell count [2]. Late presentation is associated with higher rates of mortality due to AIDS-defining conditions, but also with an increased incidence of non-AIDS defining conditions [3, 4].

The occurrence of pulmonary abnormalities is not unusual in patients with acquired immunodeficiency syndrome (AIDS). The causes could be not only infectious diseases and malignancies. There is also a number of non-infectious and non-malignant disorders which may be clinically and radiographically similar [5]. One of these conditions is the lymphotic interstitial pneumonitis (LIP), initially described in 1966 [6]. LIP is a rare benign polyclonal lymphoproliferative disorder of the lung parenchyma due to infiltration of the interstitium and alveolar spaces by lymphocytes, plasma cells, and other lymphoreticular elements [7]. It is described with an increasing frequency among HIV infected patients, especially in black African and Afro-Caribbean [8]. In HIV-associated LIP a possible mechanism is the HIV induced proliferation of bronchus-associated lymphoid tissue (BALT) [9]. Additionally, LIP is associated with autoimmune disorders such as Sjogren’s syndrome, systemic lupus erythematosus (SLE), Hashimoto's disease and autoimmune haemolytic anaemia [10, 11]. The presence of Epstein-Barr virus (EBV) Deoxyribonucleic acid (DNA) in lung biopsy specimens from HIV infected children with lymphocytic interstitial pneumonitis may demonstrate another possible etiological factor [8]. However, the aetiology of LIP remains unknown. The clinical and radiographic features are not well characterized. Thus, the differential diagnosis from infectious diseases such as pneumocystis jiroveci pneumonia could be difficult. High resolution computed tomography and transbronchial biopsy are basic tools for diagnosis [7].

The aim of the present case presentation is to report the diagnostic approach and clinical progress of HIV-associated LIP in a newly diagnosed late presenter in our HIV Unit of a tertiary University Hospital in a rural region of Greece (East Macedonia and Thrace). This case presentation highlights the importance of timely diagnosis and initiation of antiretroviral therapy. The viral suppression is a vital step in order to end the HIV epidemic and reduce the frequency of AIDS-defining conditions and other various diseases which aggravate the prognosis of patients.

## Case presentation

A 36 year old Greek male was hospitalized in March 2019 in the Infectious Diseases Unit of University Hospital of Alexandroupolis (Thrace, Greece) for persistent fever, non-productive cough, dyspnea, weight loss and fatigue. These symptoms were firstly occurred three months before the hospitalization. The findings of clinical examination were fever, tachypnea, dyspnea, reduction of breath sounds bilaterally, generalized lymphadenopathy, oral candidiasis and moderate palpable liver. His oxygen saturation was 96%. From the laboratory test a low count of red blood cells, white blood cells and platelets were revealed. The chest X-ray was normal. He had a hospitalization for bacterial pneumonia in his past medical history. The patient does not smoke and consume drugs or alcohol.

He belongs to the HIV-associated risk group of men who have sex with men (MSM), never been checked for HIV infection. Thus, the suspicion of HIV infection was raised. It was confirmed with positive Western blot. The CD4 cell count was extremely low < 50/mm^3^ and the viral load very high (HIV-Ribonucleic acid (RNA) = 300,000 copies/mL). The patient was characterized as late presenter with advanced HIV disease at B3 CDC stage. The fact that our HIV unit is in a rural area, the fear of stigma and the limited access to check points remain the main reasons of late presentation. During the period 2008–2018 49% of newly diagnosed HIV infected patients in our clinic were late presenters, still far from UNAIDS target 90–90–90%.

Expect from HIV infection the patient was tested for other coinfections. He was negative for *Hepatitis B (HBV*) and *Hepatitis C virus infection (HCV)* and *Syphilis*. We also assessed the title of Immunoglobulin M (IgM) and Immunoglobulin G (IgG) antibodies against *Toxoplasma, Cytaromegalovirus (CMV)* and *Parvo B19*. The titles of IgG CMV and Parvo B19 antibodies were positive. The Tuberculosis Skin Test (PPD test) was 0 mm.

The severe immunosuppression of the patient induced the consideration of AIDS-defining conditions and the fear of immune reconstitution inflammatory syndrome (IRIS) after the initiation of antiretroviral therapy. The patient underwent a bronchoscopy which was normal. Bronchoalveolar lavage fluid (BAL) was analyzed for microbiological examination. The culture of BAL was negative for *Pneumocystis Jiroveci, Aspergillus, Cryptococcus* (India ink stain) or *Mycobacterium Avium Complex (MAC)*. It was positive for *Methicillin-Resistant Staphylococcus aureus (MRSA)* and *Candida albicans*. Additionally, the computed tomography of brain did not reveal any concerning findings.

Until patient initiates antiretroviral therapy and the CD4 cell count is increased, he began prophylaxis for pneumocystis jiroveci pneunonia with atovaquone and not with trimethoprim-sulfamethoxazole due to glucose-6-phosphate dehydrogenase (G6PD) deficiency. He also began prophylaxis for *Mycobacterium Avium Complex* with azithromycin.

**Fig. 1:**
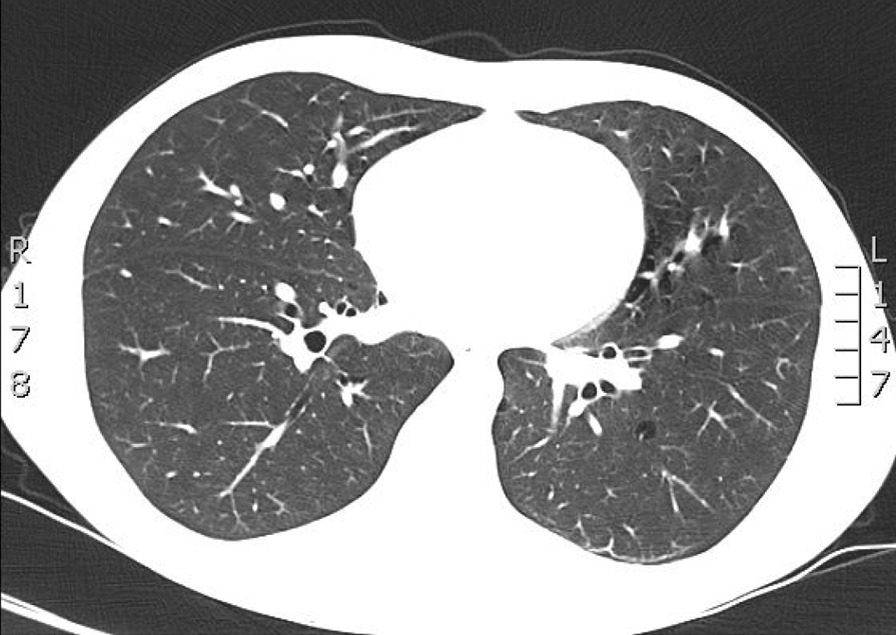
CT: multiple areas with ground-glass opacity and centrilobular nodules with a linear branching pattern (tree-in-bud sign)

The dyspnoea and non-productive cough was not improved during the hospitalization. However, his oxygen saturation and the arterial blood gas measurements were normal. After the six minutes walking test (6MWT) the oxygen saturation remained normal. The patient underwent computed tomography (CT) of the thorax in order to find out the possible cause of dyspnea. On the base of the right lower lung lobe were observed multiple areas with ground-glass opacity and centrilobular nodules with a linear branching pattern (tree-in-bud sign). Multiple bronchial cysts and diffuse interstitial infiltration were revealed in all lung lobes. The CT findings were associated with lymphotic interstitial pneumonitis (Fig. 1). Transbrochial biopsy was conducted in order to clarify the diagnosis of LIP. Diffuse interstitial cellular infiltrates by lymphocytes and plasma cells were found in interlobular and alveolar septae.

The patient initiated antiretroviral therapy (tenofovir alafenamide, emtricitabine, raltegravir) which was well tolerated. The aim is the viral suppression and the increase of CD4 cell count. Thus, the danger of AIDS-defining events will be reduced and the symptoms of LIP may improve. In the follow-up visit 3 months after the initiation of HAART patient is clinically improved without respiratory problems.

## Discussion

Lyphotic interstitial pneumonitis may be an uncommon condition, but the increased frequency in HIV infected patients indicates the need to take this diagnosis into consideration for patients with pulmonary problems. The prevalence is higher in young HIV infected women, particularly African, without excluding the diagnosis in white men like our patient [12, 13]. The most common symptoms reported are cough, dyspnea, fever, weight loss, and fatigue. A number of patients may be asymptomatic and the diagnosis is suggested due to the radiographic findings [8, 14, 15]. The clinical and radiographic findings are not specific, but computed tomography (CT) and transbronchial biopsy can be helpful for diagnosis. CT findings vary such as fine reticular or reticulonodular infiltrates in the pulmonary interstitium, coarse reticulonodular infiltrates, reticular or reticulonodular opacities with superimposed patchy alveolar infiltrates [14]. However, diagnosis cannot be suggested only by the radiographic findings. The transbronchial biopsy is important to reveal the infiltration of the interstitium and alveolar spaces by lymphocytes, plasma cells, and other lymphoreticular elements and to exclude other conditions including pneumocystis jiroveci pneumonia and tuberculosis [14].

HIV-associated lymphocytic interstitial pneumonitis has been more often described with normal CD4 cell count [8, 10]. However, the CD4 cell count in our patient when diagnosed was extremely low. The duration of symptoms at the time of diagnosis of HIV-associated LIP ranges from 1 month to several years. [8] Our patient had moderate dyspnea and non-productive cough for 3 months. The clinical condition may be progressive but in other cases may remain stable or be improved without treatment [8, 16].

The natural history of LIP is not well known inducing difficulties in the treatment. The therapeutic approach is even more difficult in HIV infected patients. Glucocorticoids have been used in various doses and duration in the past. According to the describing results improvement was induced in some cases but in a number of them the withdrawal or dose reduction led to aggravation [10, 17]. The response with antiretroviral therapy is significant probably due to viral suppression and increase of CD4 cell count [18]. Clinical and radiological improvement has been, documented only using ART, although some have been improved without ART [8, 18]. Clinical and radiological improvement has been estimated 3.5 and 6-8 months after beginning ART respectively [19].

## Conclusion

Lymphotic interstitial pneumonia is an uncommon condition with a significant frequency in people who live with HIV. LIP should be part of differential diagnosis in patients with persistent respiratory problems. Clinical and radiographic findings are not specific but high resolution computed tomography and transbronchial biopsy are helpful diagnostic tools. Our case highlights the significance of timely diagnosis and initiation of antiretroviral therapy. The increase of CD4 cell count reduces the incidence of various AIDS and non AIDS defining conditions. Treatment of the underlying HIV infection may have a beneficial effect on the symptoms and prognosis of lymphocytic interstitial pneumonitis.
